# Complementation of ELISA and an Interdigitated Electrode Surface in Gold Nanoparticle Functionalization for Effective Detection of Human Blood Clotting Defects

**DOI:** 10.1186/s11671-019-3058-z

**Published:** 2019-07-02

**Authors:** Shikui Guo, Thangavel Lakshmipriya, Subash C. B. Gopinath, Periasamy Anbu, Yaoyu Feng

**Affiliations:** 1 Department of General Surgery, The First People’s Hospital of Yunnan Province, the Affiliated Hospital of Kunming University of Science and Technology, Kunming, 650032 Yunnan China; 2 Institute of Nano Electronic Engineering, Universiti Malaysia Perlis, 01000 Kangar, Perlis Malaysia; 3 School of Bioprocess Engineering, Universiti Malaysia Perlis, 02600 Arau, Perlis Malaysia; 4 Department of Biological Engineering, College of Engineering, Inha University, Incheon, 402-751 Republic of Korea; 5 Department of Vascular Surgery, The First Affiliated Hospital of Kunming Medical University, Kumming, 650032 Yunnan China

**Keywords:** ELISA, Gold nanoparticle, Factor IX, Silanization, Blood clotting, Human serum, Interdigitated electrode

## Abstract

Developing an enhanced diagnosis using biosensors is important for the treatment of patients before disease complications arise. Improving biosensors would enable the detection of various low-abundance disease biomarkers. Efficient immobilization of probes/receptors on the sensing surface is one of the efficient ways to enhance detection. Herein, we introduced the pre-alkaline sensing surface with amine functionalization for capturing gold nanoparticle (GNP) conjugated to human blood clotting factor IX (FIX), and we demonstrated the excellent performance of the strategy. We have chosen the enzyme-linked immunosorbent assay (ELISA) and the interdigitated electrode (IDE), which are widely used, to demonstrate our method. The optimal amount for silanization has been found to be 2.5%, and 15-nm-sized GNPs are ideal and characterized. The limit of FIX detection was attained with ELISA at 100 pM with the premixed GNPs and FIX, which shows 60-fold improvement in sensitivity without biofouling, as compared to the conventional ELISA. Further, FIX was detected with higher specificity in human serum at a 1:1280 dilution, which is equivalent to 120 pM FIX. These results were complemented by the analysis on IDE, where improved detection at 25 pM was achieved, and FIX was detected in human serum at the dilution of 1:640. These optimized surfaces are useful for improving the detection of different diseases on varied sensing surfaces.

## Introduction

Improvement of all aspect at the forefront of medicine is mandatory to maintain healthy human life and to extend the lifespan. Diagnosing diseases is one of the major areas in the medical field, which should be improved further for easier identification of diseases and treatments. On the other hand, epidemic diseases such as influenza and dengue must be detected during the beginning stages to avoid spreading [1–3]. There are different diagnostics which have been demonstrated capable of genuine detection via a wide range of biomarkers [4, 5]. Among the previously generated diagnosis systems, the enzyme-linked immunosorbent assay (ELISA) is widely considered as the “gold standard” to identify the major viral and bacterial diseases and molecular species [6, 7]. Different strategies have been developed with ELISA with appealing characteristics such as ease of use, precision, and lower detection limit. In addition, the simultaneous detection of various diseases and use to screen for the major diseases is common in practice [8–10]. ELISA is an immunoassay used to detect the target of interest using the appropriate antibody on polystyrene (PS) 96-well plates. The sensitivity and selectivity of the target molecules are highly dependent on various factors in ELISA, such as the binding affinity of the target and antibody, the interaction of primary and secondary antibodies, and the efficient target immobilization on the ELISA surface. In particular, immobilization of the target on the PS plate plays a crucial role in limiting the detection. Antibody or protein may physically adsorb onto the surface of PS by the interaction of hydrophobic groups with the antibody [11]. There are some possibilities, for example, involving a weak binding of the antibody (or protein) or the instability of antibody on the PS plate and the nonuniform distribution of antibody (or protein) leading to defects in the detection. It has been proven that proper orientation of antibody on the immobilized plate improves the detection by over 64-fold as compared to the randomly immobilized molecules [12, 13]. Finding the suitable procedure for the efficient immobilization of protein or antibody on the PS plate with uniformity is thus a crucial need. Different researchers are utilizing the immobilization of proteins on PS ELISA plates by chemical or physical processes, such as a photochemical reaction assisted by polyvinyl benzyl lactonoylamide and immobilization of protein in the presence of detergent [14–16]. Dixit et al. [8] have immobilized the antibodies on the amine-modified PS plate to improve the limit of detection, and they demonstrated that the premixture of (3-aminopropyl)triethoxysilane (APTES) and antibody before the immobilization step improved sensitivity by 54 times compared with the conventional ELISA and reached the limit of detection of 10 pM [8, 17].

In the present work, we introduced a new method of protein immobilization on PS ELISA plates assisted by gold nanoparticles (GNPs). GNPs are one of the most powerful tools in the field of sensor development. They offer positive properties, such as ease of dispersion in water, biological inertness, and good compatibility with surface functionalization, and they can be tailored to uniform nanosizes [18–23]. It has been proven that GNP-conjugated antibodies improved the limit of detection with influenza virus and FIX on the waveguide-mode sensor [21–23]. GNPs are used in all kinds of sensors, including waveguide-mode sensor, surface plasmon resonance, and Raman spectroscopy for high-performance detection. Moreover, GNPs have also been used in colorimetric assays, in which the conjugation of aptamer or antibody on GNP surfaces was followed to detect the analyte by the naked eye. Researchers are also focusing on the use of GNPs in ELISA methods to enhance detection [24]. Previously, GNP-conjugated anti-mouse-IgG-HRP improved the detection of Pb(II) and reached the limit of detection of 9 pg/mL [25]. Lakshmipriya et al. have found that the conjugation of primary and secondary antibodies on GNPs improved the detection of ESAT-6 protein, which is involved in causing tuberculosis [9, 26]. Here, we used GNPs to immobilize the protein on the PS ELISA surface with the chemical modification assisted by APTES. This approach involves two steps: functionalization of the PS surface with amine groups using APTES followed by the immobilization of GNP-conjugated protein on the amine-modified PS plate. The amine groups of the APTES can bind to COOH groups on the PS ELISA surface. On the other hand, GNP-conjugated proteins stabilized with anionic citric ions can bind to the cationic APTES by electrostatic interaction [11, 27, 28]. The amino-terminal groups in the APTES displace the citrate groups on the GNPs and chemically fix onto the PS plate [29]. The positively charged amine groups in APTES also attract the negatively charged GNPs. In addition, APTES treatment changes the PS substrate to make it more hydrophobic [30].

**Fig. 1:**
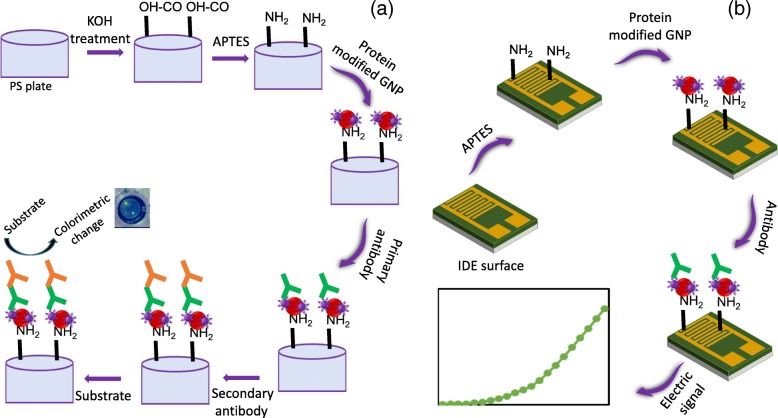
**a** Schematic representation of the GNP-assisted ELISA. GNP was premixed with protein and immobilized on APTES-modified ELISA surface. Antibodies were then added. Finally, substrate for HRP was used to detect the target. **b** Schematic representation of the GNP-assisted IDE

A chemically modified ELISA plate and GNP conjugation were used to detect factor IX (FIX) from the human blood clotting cascade. FIX is an important protein in the blood clotting system, and lower concentrations of FIX in the bloodstream lead to clotting deficiency. The enhanced immobilization of FIX on the ELISA plate through GNPs enables a potential pathway for detection of the lower levels of FIX. The GNP-modified ELISA plate showed drastic improvement in FIX detection. Furthermore, we also demonstrated the detection of FIX in human serum, and the current strategy can be used with ELISA to detect other biomarkers. To complement the above study with the ELISA substrate, we also applied the above strategy on another widely used interdigitated electrode surface (IDE) in an electrochemical dielectric sensor to detect pure FIX and FIX in a human diluted serum sample (Fig. 1a, b).

## Materials and Methods

### Reagents and Biomolecules

(3-Aminopropyl)triethoxysilane (APTES) and human serum were purchased from Sigma-Aldrich (USA). FIX protein and anti-FIX antibody were from Abcam (Malaysia). Anti-mouse-IgG-conjugated horseradish peroxidase (anti-mouse immunoglobulin HRP) was purchased from Thermo-Scientific (USA). The ELISA plate was procured from Becton Dickinson (France). ELISA 5× coating buffer was obtained from BioLegend (UK). Bovine serum albumin (BSA) and HRP substrate were obtained from Promega (USA). Gold nanoparticles (GNPs) (10, 15, and 80 nm) were obtained from Nanocs (USA). The analytical ELISA reader was from Fisher Scientific (Malaysia).

### Optimization with Silane Reagent and GNPs

To optimize the suitable concentrations of APTES and GNPs, we carried out the immobilization process with different combinations of APTES and GNPs on the ELISA plate. Initially, the ELISA plate was hydroxylated with 1% KOH for 1 h. Then, 1.25, 2.5, and 5% APTES were linked on separate wells of the ELISA plate at room temperature (RT) for 5 h. After that, the diluted GNPs (in distilled water) with 25, 50, and 100% concentration were immobilized on the APTES-modified surfaces. FIX, at 200 nM, was then allowed to bind onto the GNP surfaces. The remaining binding sites were then blocked using 2% BSA, then a 1:1000 dilution of FIX antibody was introduced, followed by the addition of a 1:1000 dilution of anti-mouse-IgG-HRP. Finally, the substrate was added to detect FIX interaction. The wells were washed 5 times between each step using washing buffer (PBS buffer containing 0.05% Tween 20, pH 7.4). Finally, the optical density (OD) was measured by ELISA reader at 405 nm. GNPs of 15 nm size were used in this experiment.

### Determining the Necessary Size of GNPs

After determining the suitable concentration of APTES and GNP dilution under the optimal conditions, 10, 15, and 80 nm GNPs were tried for FIX immobilization to determine the most suitable size. The same experimental conditions were used as stated above. APTES at 2.5% and a GNP dilution of 50% were used for the FIX immobilization.

### Specific and Selective Detection of FIX

We also confirmed the specific binding of protein and antibody on the ELISA plate. For that, we utilized three different kinds of control experiment. We immobilized the following order of biomolecules on the ELISA plate to serve as controls and specific conditions: control 1 (C1), FIX-BSA-anti-mouse IgG-substrate; control 2 (C2), FIX-BSA-FIX antibody-substrate; control 3 (C3), BSA-FIX antibody-anti mouse IgG-substrate, specific (S), FIX-BSA-FIX antibody-anti-mouse IgG-substrate.

### Detection of GNP-Conjugated FIX on ELISA Plate vs. Conventional ELISA

FIX was detected using the GNP-modified ELISA plate by two different methods, such as APTES-GNP-FIX and APTES-GNP premixed FIX, and these methods were compared with the conventional ELISA. The following steps constitute the procedures. Conventional ELISA: (i) FIX protein was diluted in 1× coating buffer with concentrations from 0 to 200 nM, (ii) blocked with 2% BSA, (iii) 1:1000 dilution of FIX antibody was added, (iv) 1:1000 dilution of anti-mouse-IgG-HRP was added, (v) substrate for HRP was added and measured at 405 nm.

APTES-GNP-FIX (method 1): (i) PS plate was activated by 1% KOH, (ii) 2.5% APTES was added onto the ELISA plate at RT for 5 h, (iii) 25% of the received 15 nm GNP was added and kept at 4 °C overnight, (iv) FIX with concentrations from 0 to 200 nM was allowed to bind independently onto the GNP surface, (v) the remaining surface was blocked by 2% BSA, (vi) 1:1000 dilution of FIX antibody was added, (vii) 1:1000 dilution of anti-mouse-IgG-HRP was added, (vii) substrate for HRP was added and measured at 405 nm.

APTES-GNP premixed FIX (method 2): (i) PS plate was activated by 1% KOH, (ii) 2.5% APTES was added at RT for 5 h, (iii) premix of 25% 15 nm GNP solutions with different concentrations of FIX (incubated at RT for 30 min; 0 to 200 nM) were immobilized on the APTES-modified surface, (iv) the remaining surface was blocked by 2% BSA, (v) 1:1000 dilution of FIX antibody was added, (vi) 1:1000 dilution of anti-mouse-IgG-HRP was added, (vii) substrate for HRP was added and measured at 405 nm.

### Detection of FIX in Human Serum and After Spiking FIX

To detect FIX in human serum, we premixed the serum with GNPs instead of FIX here. Serum dilutions from 20 to 1280 were titrated and mixed with GNPs to evaluate the detection of FIX. Other experimental conditions and concentrations were used, similarly to the previous experiments.

Spiking experimentation was conducted by spiking different concentrations of FIX (0 to 8 nM) into the optimized dilution of human serum and premixing with GNPs before immobilization on the ELISA plate. Other experimental conditions and concentrations were used as in the previous experiments.

### Interdigitated Electrode Surface Fabrication

The interdigitated electrode (IDE) surface fabrication process includes wafer cleaning, oxide layer growth, negative photoresist spin coating, photoresist patterning, aluminum metal deposition, photoresist removing, and wafer dicing. The process was initiated on buffered oxide etchant cleaned wafer followed by the oxidation on the silicon wafers (310 nm) at high temperature (1000 °C), then the etching process was performed using aluminum. Negative photoresist was deposited by spin coater with a spin speed of 2000 rpm. The resist was developed by RD–6 after exposure to UV light (240 s). Then, 240 nm aluminum was deposited by a thermal evaporator (Edwards Auto 306) at 3.0E−5 Torr. The photoresist was stripped using acetone until solid IDE appeared, then diced to create single IDE. Finally, the zinc oxide layer was overlaid on the top for ultimate biomolecular immobilization [31]. Before proceeding with zinc oxide attachment, the sensing surface was initially washed with 1 M potassium hydroxide (pH 9.0).

### Detection of FIX on a GNP-Modified Surface

After the optimization and confirmation of detection efficiency of FIX with GNP-modified ELISA substrate, the same surface modification was performed on the IDE surface to complement the above detection. Initially, IDE zinc oxide surface was modified with amine by adding 2.5% APTES diluted in ethanol for 3 h at RT. After that, the surface was washed with ethanol five times, and the premix of GNPs (25%) and FIX was added. Then, the remaining surfaces were blocked by ethanolamine, and the FIX antibody was added next to detect FIX. The concomitant changes in the current signal were noticed. To check the limit of detection, different concentrations of FIX were mixed independently with GNPs, immobilized on the amine-modified IDE surface, and then detected by the antibody; the limit of detection was determined by 3σ calculation. The abundance of FIX in the diluted human serum mixed with GNPs was immobilized on the amine-modified IDE surface and then detected by its antibody. The fabrication of the above surface followed as stated before [31].

## Results and Discussion

### Pretreatment on a PS ELISA Surface

Effective immobilization of protein or antibody on the polystyrene (PS) ELISA surface apparently improves the detection limit of the target-probe interaction. Different immobilization methods have been used for protein or antibody attachment on the ELISA plate. In this work, we introduced a chemically modified ELISA surface with GNPs for protein or antibody immobilization. We desired to use one of the important clotting factors, such as factor IX (FIX), which has been widely reported to be important [32–36]. Figure 1 shows the schematic representation of the protein immobilization on the polystyrene ELISA surface. First, the ELISA surface was activated by 1% KOH. In the absence of KOH pretreatment, the number of APTES molecules to be bound on the PS surface is minimal. This is because of the lack of polar and hydrogen bond accepting groups, which facilitate the initial adsorption of APTES on polymers [11]. After KOH treatment, the surface was modified with an amine by the appropriate concentration of APTES in order to capture the GNPs.

### Characterization of GNPs

**Fig. 2:**
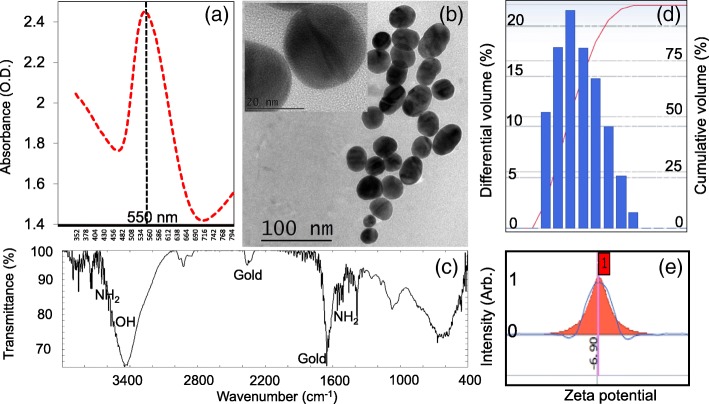
Characterization of gold nanoparticles. Performed with the optimal size 15 nm. **a** UV-Vis spectrophotometry analysis. A prominent single peak was found at 550 nm. **b** Transmission electron microscope observation. Figure inset shows the enlarged image. Scale bar is shown. **c** Fourier-transform infrared spectroscopy analysis. Apparent peak positions for gold and NH_2_ are indicated. **d** Volume distribution analysis of GNP. The obtained distribution is shown. **e** Zeta potential analysis. The value was found to be − 6.9

Before proceeding further, we characterized the as-received GNPs (15 nm) by optical, morphological, and size-distribution measurements. Figure 2a displays the UV-Vis spectrophotometry analysis of the GNPs, which apparently displayed the peak maxima at 550 nm. Transmission electron microscope observations show the size of the GNPs to be ~ 15 nm and the shape to be good (Fig. 2b and inset). Fourier transform infrared spectroscopy analysis was performed from the wavenumber of 500 until 4000 cm^−1^ (Fig. 2c). An apparent single peak at 1650 cm^−1^ was noticed for the gold along with another minor peak, whereas the presence of peaks for amine groups representing the attachment of APTES. Further support was rendered by the volume distribution and zeta potential analyses. Based on this analysis, the volume distribution indicates that there is a slight variation in the size distribution (Fig. 2d) and that the zeta potential was − 6.9, representing the stability of the GNPs used in this study (Fig. 2e).

### Preparation and Optimization of Amine Surface to Capture GNPs

**Fig. 3:**
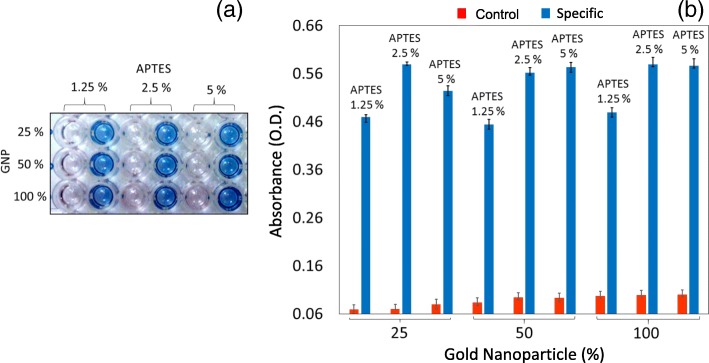
Determining the amounts of APTES and GNP. Three different concentrations (1.25, 2.5, and 5%) of APTES were used with three dilutions of GNP (25, 50, and 100%). **a** Visual detection of FIX after adding the substrate. Control lanes without FIX were included. **b** Graphical representation of the optimum APTES level. 2.5% APTES and 25% GNP were found to be optimal

The amount of APTES greatly influences the immobilization of GNP or antibody on the PS surface. In general, highly crowded APTES molecules have a probability of creating false-positive results. Since APTES has been used from 1 to 2% to immobilize the antibody in several sensor surfaces, here, we titrated from 1.25 to 5% APTES, prepared in absolute ethanol. Next, our aim was to immobilize GNPs on the APTES-modified surfaces. There is a possibility for the higher numbers of GNPs to yield the crowding effect, so GNPs with 25, 50, or 100% of 15 nm were tested on the APTES-modified surface to optimize the suitable dilution. On these modified surfaces, 250 nM of the FIX was detected with FIX antibody. As shown in Fig. 2a and b, 1.25% APTES shows less optical absorbance (OD) with all dilutions of GNPs (25, 50, and 100%), whereas 2.5 and 5% APTES showed higher absorbance with both 50 and 100% of GNPs. At the same time, the signal-to-noise ratio was improved with increasing APTES and GNP concentrations (Fig. 3a, b). The 5% APTES with 50 and 100% GNPs showed nonspecific binding, and 50 and 100% GNPs with 2.5% APTES displayed similar absorbance. Based on these obtained values, we chose 2.5% APTES and 25% dilution of GNPs for further experiments.

### Determination of potential GNP size

**Fig. 4:**
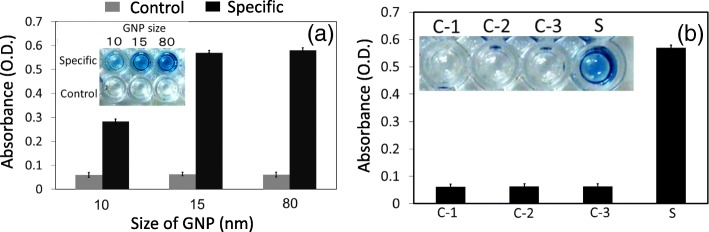
**a** Determining the ideal size of GNP. Among 10, 15, and 80 nm, 15 and 80 nm showed similar results. Fifteen nanometers was used to determine the limit of detection with FIX. **b** A control experiment for the detection of FIX. Three different control experiments (C1—without FIX antibody; C2—without anti-mouse IgG; C3—without FIX) were conducted. No significant signal was found in any of the control experiments. Figure insets display the visual results

After APTES and GNP dilution optimization, we next evaluated the suitable size of GNPs. Since the surface area plays a crucial role in protein immobilization, here, we tried to immobilize three different sizes of GNPs, including 10, 15, and 80 nm. Different sizes of GNPs have different surface areas and different charges to attract the protein, and the binding capability on the APTES-modified surface is also different. As shown in Fig. 4a, 250 nM FIX was detected with the ELISA surface modified with 10, 15, and 80 nm GNPs. It was found that 15 and 80 nm GNPs show almost the same absorbance of 0.4 (OD) for the detection of 250 nM of FIX, but 10-nm-sized GNPs show only 0.34 OD. From this experiment, we confirmed that 15 or 80 nm GNPs are suitable for further experiments, and we continued with 15 nm GNPs.

### Experimental strategies to confirm nonfouling and high performance

Before detecting the FIX on the GNP-modified ELISA surface, we performed three different kinds of control experiment as explained in the “Materials and Methods” section. As shown in Fig. 4b, we could not observe significant absorbance in the wells for control experiments 1, 2, and 3 (C1, C2, and C3). In the specific experiment (S), the higher level of absorbance is clearly associated with apparent color changes. In the case of C1, we did not add FIX antibody, which confirms that FIX antibody specifically interacts with FIX (in S). No observable signal was found in the absence of anti-mouse IgG in C2, which gives further anchorage for the proper interaction of FIX and antibody followed by anti-mouse IgG. In C3, in the absence of FIX, we could not measure the significant absorbance. From these results, the proper interaction of FIX with its antibody on the ELISA surface was confirmed without nonspecific binding.

### GNP-Assisted ELISA vs. Conventional ELISA: Determination of Sensitivity

**Fig. 5:**
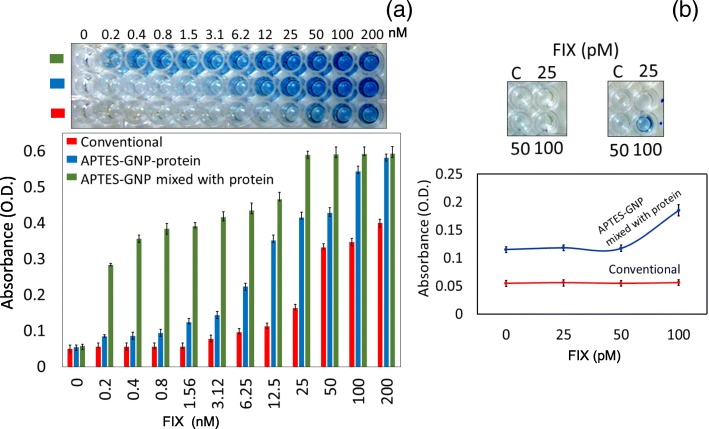
**a** Comparing the sensitivity of FIX in GNP-modified ELISA against the conventional ELISA. FIX was titrated from 0.2 to 200 nM. Conventional ELISA exhibits the limit of detection of 6 nM. On the GNP-modified ELISA, the signal was observed until 200 pM. Figure inset displays the visual results. **b** Limit of detection of FIX on GNP-modified ELISA plate. FIX was titrated from 25 to 100 pM. The limit of detection was found to be 100 pM. Figure insets display the visual results

After carrying out the control experiments, we detected FIX with GNP-modified surfaces and compared the results with the conventional ELISA method. We conducted three different experiments for the detection of FIX. First, GNP was immobilized on APTES-modified surfaces, and then FIX was attached onto the surface of GNP through electrostatic interaction. Due to the electrostatic interaction, most of the proteins have the possibility to bind onto the GNP surface; in that way, we could immobilize FIX on the ELISA PS surface. The protein immobilization also depends on the charges arising from the amino acids and GNP. Gopinath et al. [37] have proved the strong binding of FIX on the GNP surface, and they also found that it is stable under high salt conditions. In the second method, GNP and protein were first premixed before being immobilized on the aminated ELISA PS surface. It was expected that more proteins can bind in this way, because there is a higher possibility that protein molecules are able to bind onto the GNP surface in the solution state. The premixed solution was then immobilized on the APTES-modified amine surfaces. These methods were compared with the conventional ELISA method. As shown in Fig. 5a, when we titrated FIX concentration from 0 to 200 nM, conventional ELISA (method 1) showed the limit of detection of 6 nM. The APTES-GNP-FIX method (method 2) displays the limit of detection of 200 pM, which is over 30 times higher sensitivity compared with the conventional ELISA. Sensitivity enhancement is achieved through proper orientation of high numbers of antigens immobilized on the ELISA PS plate, and ultimately more antibodies will interact. In this research, gold nanoparticles were used to immobilize more antigens through amine modification of the ELISA plate. In the conventional ELISA, antigens are directly immobilized on the ELISA plate, which leads to lesser numbers of molecules binding on the surface and causes reduced sensitivity versus the current gold nanoparticle-mediated strategy. In addition, localized surface plasmon resonance gives further sensitivity improvement, as revealed in the studies with GNPs. When we premix GNPs with FIX, as expected, the limit of detection was highly improved and attained the level of 100 pM, which is 60 times higher sensitivity than conventional ELISA. As shown in Fig. 5a, it could be clearly seen that when we use GNPs for protein immobilization, the maximum absorbance reached ~ 0.6 for both premixed and directly immobilized proteins on the GNP surfaces, but the absorbance found with conventional ELISA was ~ 0.4 using the similar concentration (200 nM) of FIX. Even with other concentrations, method 3 showed a higher difference in absorbance compared to the conventional ELISA, and this was also reasonably higher than the method 2. In the case of conventional ELISA, the visible detection was found from 25 nM, and from 3 nM in method 2. With method 3, we could visibly see the color change from 200 pM of FIX because of higher absorbance (Fig. 5a). The absorbance was saturated at 25 nM in method 3. Since we could visualize the color changes from 200 pM of FIX, we evaluated the limit of detection and titrated using the lower concentrations of FIX. It was apparent that the detection limit reached 100 pM when we premixed the GNPs and FIX (Fig. 5b).

### Detection of FIX in Human Serum and Enhancement by Spiking FIX

**Fig. 6:**
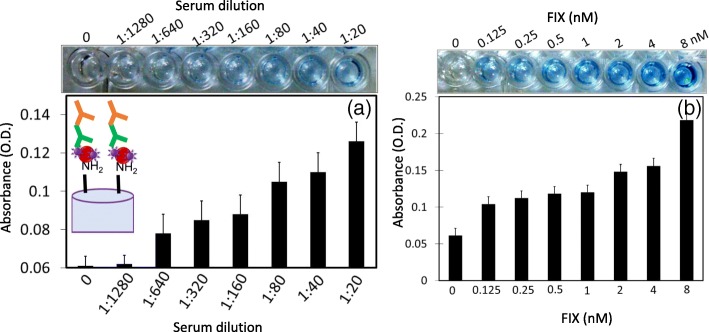
**a** Detection of FIX in human serum. Human serum was diluted from 1:20 to 1:1280. FIX was detected in human serum diluted 1:640 (figure inside indicates the schematic representation). **b** Spiking of FIX (0.125 to 8 nM) into 1:640 dilution of human serum. With increments in the FIX concentration, the absorbance was increased. Figure insets display the visual results

Since FIX was found to play an important role in the clotting cascade [38, 39], after checking the limit of detection, we also detected FIX in human serum. For this analysis, we titrated the human serum in the range between 1:1280 and 1:20 dilutions. The diluted serum was premixed with GNPs and immobilized on the APTES-modified ELISA surface, as stated above. It was obvious that the FIX was detected from 1:640 dilution of serum, which is equivalent to 125 pM FIX (Fig. 6a). The healthy human serum usually contains approximately 87 nM FIX, along with other major proteins such as albumin and globulin. With our strategy, we reached the specific detection limit of 125 pM in human serum, which is useful for identifying a blood clotting defect. On the other hand, when we spiked FIX from 1 to 8 nM into the 1:640 dilution of human serum, it displayed increments in the absorbance which concomitantly increased with FIX concentrations (Fig. 6b).

### Detection of GNP-FIX on IDE Surface: Complementation Analysis

**Fig. 7:**
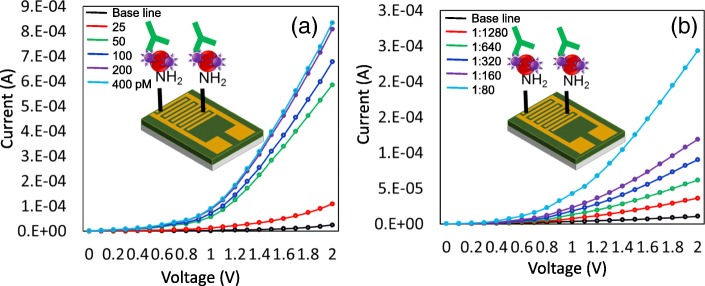
**a** Limit of detection of FIX-conjugated GNP on IDE sensing surface. FIX was titrated from 25 to 400 pM. The limit of detection was found to be 25 pM. **b** Detection of FIX in human serum on the IDE sensing surface. Human serum was diluted from 1:80 to 1:1280. FIX was detected in human serum diluted 1:1280

Since it was found that GNP-conjugated FIX improved the detection limit, we applied a similar strategy using the electrochemical sensor with IDE surface to complement these findings. We titrated the FIX from 25 to 200 pM (mixed with GNPs), immobilized it on the amine-modified IDE surface, and then detected it by FIX antibody. As shown in Fig. 7a, with increase in FIX concentration, the current level was also increased concomitantly from the baseline. FIX level was saturated from 200 pM, and the limit of detection was found to be 25 pM. This detection is fourfold enhanced compared with ELISA (100 pM). In addition, FIX detection was evidenced in the diluted human serum (Fig. 7b). For this experiment, human serum was diluted from 1:80 to 1:1280. As shown in the figure, the dilution of 1:1280 (red curve) shows an apparent difference from the baseline (black curve) with the 3σ estimation, indicating that the evident detection of FIX from human serum with the dilution of 1:1280 matches with the results from ELISA (detected at 1:640). Detection from IDE with lesser dilution shows the increment in the sensitivity.

## Conclusion

Efficient immobilization of protein or antibody on the PS ELISA surface is a crucial step to improve the limit of detection. Here, we introduced a new and easy protein immobilization strategy on the PS ELISA surface assisted by GNPs. Two different methods were used: one is the direct immobilization of FIX on the GNP-modified surface, and another one is premixing of FIX with GNPs before attachment. It was found with the former method that improvement of the limit detection is 30-fold higher compared with the conventional ELISA. The latter method showed a 60-fold improved limit of detection compared to the conventional ELISA. We also demonstrated the detection of FIX in 1:650 diluted human serum, which is equivalent to 125 pM. Obviously, these strategies proved that the higher binding of proteins on the ELISA surface was able to improve the sensitivity and to be useful for detecting a wide range of biomarkers on the ELISA surface.

## Data Availability

All of the data are fully available without restriction.

## Abbreviations

APTES: (3-Aminopropyl)triethoxysilane
BSA: Bovine serum albumin
COOH: Carboxyl
ELISA: Enzyme-linked immunosorbent assay
Fc: Fragment crystallizable
GNP: Gold nanoparticle
HRP: Horseradish peroxidase
IDE: Interdigitated electrode
IgG: Immunoglobulin
OD: Optical density
PEG: Polyethylene glycol
PS: Polystyrene
PVLA: Polyvinyl benzyl lactonoylamide
TMB: 3,3′,5,5′-Tetramethylbenzidine
